# P-572. Real-World Experience with Long-Acting Injectable Cabotegravir/Rilpivirine (CAB/RPV LA) in an Academic Medical Center

**DOI:** 10.1093/ofid/ofae631.770

**Published:** 2025-01-29

**Authors:** Oleksandr Semeniuk, Alexander Ogilvy, Maria Elena Ruiz, Jose A Lucar, Afsoon Roberts, Chelsea Ware, Marc O Siegel

**Affiliations:** The George Washington Medical Faculty Associates, Washington, District of Columbia; The George Washington Medical Faculty Associates, Washington, District of Columbia; GWU, Washington DC, District of Columbia; George Washington University, Washington, District of Columbia; George Washington University, Washington, District of Columbia; The GW Medical Faculty Associates, Washington, DC; George Washington University School of Medicine and Health Sciences, Washington, District of Columbia

## Abstract

**Background:**

Since the approval of CAB/RPV LA, over 50 patients followed in the MFA Infectious Diseases clinic have transitioned to long-acting injectable CAB/RPV. Several patients were found to have low-level viremia despite no documented resistance to either component of CAB/RPV LA. We investigated the incidence of low-level viremia and associated risk factors in this patient population.

**Methods:**

We reviewed the charts of all patients who received at least 3 doses of CAB/RPV LA and evaluated the incidence of HIV viral loads (VL) ≥50 copies. Those patients were categorized into 3 groups: a) blips – isolated VL of 51-200 copies that resolved on subsequent test, b) low-level viremia – sustained VL of 51-200 copies on consecutive tests, c) virologic failure – VL over 200 copies. For all patients, we reviewed age, gender, race, weight, BMI, HIV VL, CD4 count/CD4% before initiating CAB/RPV LA, prior antiretroviral therapy, history of prior resistance mutations, comorbid conditions (diabetes, kidney disease, chronic hepatitis, cancer), tobacco use, and statin use.

**Results:**

51 patients met inclusion criteria for the study. Seven patients (13.7%) had blips, one patient (2%) had low-level viremia, and one patient (2%) had evidence of virologic failure, although this was not sustained on repeat testing. Four patients discontinued treatment due to side effects or social reasons. The only statistically significant risk factor for subsequent viremia was the presence of viremia prior to initiation of CAB/RPV LA: 2.7 % (1/37) in the group with no VL≥50 after the initiation of therapy vs. 33.3% (3/9) in the group that later had blips or viremia, p=0.02. There was also a tendency to the higher incidence of viremia in patients with diabetes mellitus although it did not reach statistical significance (7.1% vs. 33.4%, P=0.06).

**Conclusion:**

CAB/RPV LA is a valuable option for HIV management in the outpatient setting. We documented a relatively small incidence of viral blips and low-level viremia in our population, with statistical significance seen in those with detectable viremia at initiation of CAB/RPV LA. Of the patients included in the analysis, there was no sustained virologic failure confirmed by viral load retest and resistance testing.

**Disclosures:**

**All Authors**: No reported disclosures

